# A national clinical decision support infrastructure to enable the widespread and consistent practice of genomic and personalized medicine

**DOI:** 10.1186/1472-6947-9-17

**Published:** 2009-03-23

**Authors:** Kensaku Kawamoto, David F Lobach, Huntington F Willard, Geoffrey S Ginsburg

**Affiliations:** 1 Division of Clinical Informatics, Department of Community and Family Medicine, Box 104007, Duke University Medical Center, Durham, North Carolina 27710, USA; 2 Duke Institute for Genome Sciences & Policy, Duke University, Durham, North Carolina, USA

## Abstract

**Background:**

In recent years, the completion of the Human Genome Project and other rapid advances in genomics have led to increasing anticipation of an era of genomic and personalized medicine, in which an individual's health is optimized through the use of all available patient data, including data on the individual's genome and its downstream products. Genomic and personalized medicine could transform healthcare systems and catalyze significant reductions in morbidity, mortality, and overall healthcare costs.

**Discussion:**

Critical to the achievement of more efficient and effective healthcare enabled by genomics is the establishment of a robust, nationwide clinical decision support infrastructure that assists clinicians in their use of genomic assays to guide disease prevention, diagnosis, and therapy. Requisite components of this infrastructure include the standardized representation of genomic and non-genomic patient data across health information systems; centrally managed repositories of computer-processable medical knowledge; and standardized approaches for applying these knowledge resources against patient data to generate and deliver patient-specific care recommendations. Here, we provide recommendations for establishing a national decision support infrastructure for genomic and personalized medicine that fulfills these needs, leverages existing resources, and is aligned with the *Roadmap for National Action on Clinical Decision Support *commissioned by the U.S. Office of the National Coordinator for Health Information Technology. Critical to the establishment of this infrastructure will be strong leadership and substantial funding from the federal government.

**Summary:**

A national clinical decision support infrastructure will be required for reaping the full benefits of genomic and personalized medicine. Essential components of this infrastructure include standards for data representation; centrally managed knowledge repositories; and standardized approaches for leveraging these knowledge repositories to generate patient-specific care recommendations at the point of care.

## Background

### The Promise of Genomic and Personalized Medicine

Through the 1990s and into the current millennium, rapid advances in genomics and related disciplines have made it possible to envision a healthcare system in which patient care is routinely optimized through the use of information on individuals' genomes and their downstream products (i.e., transcriptomes, proteomes, and metabolomes). Genomic medicine, or the use of genomic information to optimize health and healthcare [1], is a critical component of personalized medicine, which entails the optimization of individuals' health through a consideration of all available patient data, including molecular data on patients' genomes, transcriptomes, proteomes, and metabolomes [1,2]. Because variations in individuals' genetic profiles oftentimes correlate with differences in how individuals develop diseases and respond to treatment, personalized medicine supported by genetic and genomic assays has the potential to facilitate optimal risk identification, disease screening, disease diagnosis, therapy, and monitoring [1,2].

In addition to genomic assays, proteomic and metabolomic signatures hold great potential for serving as pillars of personalized medicine in the future [3-7]. At present, however, genomic assays (including gene expression profiling and genome sequence analysis) appear better poised than proteomic and metabolomic signatures for overcoming the technical, scientific, and logistical barriers to achieving widespread clinical use in the near future [4-6,8]. Thus, our discussion of personalized medicine in this manuscript focuses primarily on the clinical use of genetic and genomic assays.

While personalized medicine guided by genomics is still in early stages of development, individuals' genetic profiles are already starting to be used to guide patient care. As some examples, clinicians can obtain gene expression profiles of breast cancer samples to guide management [9], genotypes of HIV samples to identify the optimal antiretroviral regimen [10], and genetic profiles of patients' cytochrome P450 drug metabolizing system to guide the selection and dosing of pharmacotherapies [11]. In the coming years, we expect that many more validated genetic and genomic assays will become clinically available for guiding patient management. In particular, given the significant role of genetic variability in determining how patients respond to pharmacotherapy [12], validated genetic and genomic tests may soon be available for guiding the pharmacotherapy of a variety of common diseases including asthma [13], hyperlipidemia [14], hypertension [15], and various cancers [16]. By enabling individually tailored medical interventions associated with improved effectiveness, reduced side effects, and greater patient compliance, genetic and genomic assays could transform healthcare systems and lead to significant reductions in morbidity, mortality, and overall healthcare costs [1,2].

### Challenges to Genomic and Personalized Medicine

Despite the great promise of the clinical use of genomics, many obstacles lie in the way of integrating genomic and personalized medicine into routine clinical care. These challenges include the need for greater oversight and quality assurance of genetic testing [17]; limited availability of rigorous, prospectively collected evidence on the clinical value and cost effectiveness of genetic and genomic assays [17-22]; concern among pharmaceutical companies that the tailoring of pharmaceuticals to genetic sub-populations may segment the marketplace and reduce the number of patients for whom a given medication is indicated and prescribed [21]; and the limited knowledge of, and comfort with, genetics and genomics among healthcare professionals [17,19,22,23].

In this manuscript, we focus on the need for a robust health information technology (IT) infrastructure supportive of appropriate clinical decision making based on the results of genetic and genomic assays combined with other clinical data. Of note, this need has been identified as a critical requirement for realizing the promise of personalized medicine by the U.S. Secretary of Health and Human Services [24] as well as by others [21,25]. However, it is important to keep in mind that the need for a decision support infrastructure represents just one of several substantial obstacles that must be overcome before genomic and personalized medicine can become routine components of clinical care.

### Need for Clinical Decision Support

Despite the potential for genomics to transform healthcare, past experience indicates that new genomic interventions, like any new medical intervention, will remain substantially underutilized for many years unless a robust infrastructure is established for supporting their appropriate use. Clinical research results require an average of 17 years to be routinely implemented in clinical practice [26], and a landmark 2003 study assessing 439 quality indicators found that U.S. adults only receive about half of recommended care [27]. Moreover, genomic interventions may face even greater barriers to clinical adoption compared to more traditional medical interventions, due to such factors as limited clinician familiarity with genomics [23] and the volume and complexity of the underlying data that may need to be considered.

Of the many strategies that have been evaluated for promoting evidence-based care, one strategy has been found to be particularly effective: clinical decision support (CDS), which entails providing clinicians, patients, and other healthcare stakeholders with pertinent knowledge and/or person-specific information, intelligently filtered or presented at appropriate times, to enhance health and healthcare [28]. Over 90% of clinician-directed CDS interventions evaluated in randomized controlled trials have significantly improved patient care, provided that the CDS was delivered automatically as a part of clinician workflow, offered at the time and location of decision making, recommended a specific course of action, and used a computer to generate the recommendation [29].

Extended to genomic and personalized medicine, CDS could support the consistent and evidence-based application of genetic and genomic information in healthcare in many ways. For example, when initiating warfarin therapy using an electronic prescribing system, a clinician could be provided with recommendations on dosing and monitoring that account for the patient's CYP2C9 and VKORC1 genotype [30]. As another example, to support a clinician in his treatment of a patient with breast cancer, an electronic health record (EHR) system could consider the gene expression profile of the patient's cancer biopsy and provide an individually-tailored prediction of how the patient is likely to respond to various therapeutic options [9]. In championing this vision, Secretary Leavitt of the U.S. Department of Health and Human Services has identified the enabling of personalized healthcare through health IT a priority area of the department [24]. Moreover, the Secretary's Advisory Committee on Genetics, Health, and Society has identified CDS as an important component of the health IT infrastructure required for the widespread and effective practice of personalized medicine [25].

Critical to this vision of genomic and personalized medicine supported by IT will be a national CDS infrastructure that enables authoritative, centrally-curated knowledge on genomic medicine to be consistently leveraged in clinical practices across the nation. In this manuscript, we define the essential components of this infrastructure, explain why each component is needed, and provide recommendations for establishing this infrastructure. While we focus primarily on the needs facing the U.S. healthcare system, we anticipate that our recommendations will be helpful to the healthcare stakeholders of any nation seeking to reap the full benefits of genomic and personalized medicine.

## Discussion

### Required Infrastructure Components

In order to provide CDS, several prerequisites must be met. First, there must be medical knowledge available on how patients should be managed, and that knowledge must be encoded in a format that can be processed by a computer to generate patient-specific advice in an automated manner. Second, the patient data required for generating these inferences must be available in a format that can be interpreted by a computer. Finally, a mechanism must be in place for applying the computer-processable medical knowledge against the patient data to generate patient-specific assessments and recommendations. This advice can then be communicated to clinicians, patients, and other healthcare stakeholders to guide their decision making.

A national CDS infrastructure is required for genomic and personalized medicine because the CDS prerequisites just described cannot be efficiently fulfilled unless certain infrastructure components are in place. These required infrastructure components are outlined in Table 1. Of note, these infrastructure needs exist not only for genomic and personalized medicine but for all aspects of traditional clinical care as well. Due in large part to the lack of these infrastructure components, only limited CDS capabilities are currently available for supporting traditional medical care in the vast majority of healthcare settings, if at all [28]. This need for a national CDS infrastructure will only be exacerbated as the number of genomics-based clinical management algorithms increases rapidly, and perhaps exponentially, in the coming years.

**Table 1:** Infrastructure components required for meeting CDS prerequisites

CDS Prerequisite	Needed Infrastructure Component	Reason for Need
Computer-processable medical knowledge	Centrally managed repositories of computer-processable medical knowledge	Most healthcare organizations lack the resources to create and maintain required knowledge repositories for themselves
	Standardization of CDS information provided for specific aspects of genomic medicine	All knowledge resources should provide consistent CDS information for common aspects of genomic medicine

Computer-interpretable patient data	Standardized representation of patient data	Variable representation of patient data makes CDS resources difficult to re-use across different settings

Generation of patient-specific advice using knowledge and patient data	Standard approaches for leveraging computer-processable medical knowledge	Disparate approaches to integrating knowledge resources make such resources difficult to re-use across different settings
	Standard approach for locating and retrieving patient data	Patients receive care across institutional boundaries

One critical component of a national CDS infrastructure is centrally managed repositories of authoritative, computer-processable knowledge on how genetic and genomic interventions should be utilized clinically. Knowledge resources included in these repositories may consist of two broad types of resources: knowledge resources that encapsulate only the core medical knowledge underlying a clinical decision (e.g., knowledge on how to calculate the optimal warfarin initiation dose given a patient's phenotype and genotype [30]), and knowledge resources that encapsulate both the core medical knowledge as well as additional business logic on how the resource should be utilized in a specific setting (e.g., when a warfarin order is placed with a computerized provider order entry system, relevant clinical and genetic parameters should be collected from the EHR and from the ordering clinician; the optimal warfarin dose should then be calculated; and the calculated dose should be presented to the clinician as the default ordering dose along with an explanation) [31]. For the purposes of this manuscript, the former type of knowledge resource will be referred to as context-independent knowledge resources, and the latter type will be referred to as context-inclusive knowledge resources. A context-inclusive knowledge resource is easier to utilize when the resource is being used in the context in which it was intended (e.g., the order entry system of a specific vendor), whereas a context-independent knowledge resource is easier to utilize when the resource is being leveraged in a different setting. Of note, a context-independent knowledge resource may be leveraged to generate one or more context-inclusive knowledge resources tailored to specific use contexts.

Because the task of creating and maintaining computer-processable medical knowledge requires significant expertise and effort, most healthcare organizations lack the resources to create and maintain these knowledge repositories for themselves. Thus, centrally managed repositories of computer-processable knowledge are needed to enable CDS for genomic and personalized medicine on a widespread basis. Organizations that could lead this knowledge management process include academic medical centers, professional associations, governmental agencies, and commercial entities. With regard to the potential role for corporations, companies such as First DataBank [32], Thomson Reuters^® ^[33], Elsevier^® ^[34], Wolters Kluwer^® ^Health [35], and Zynx^® ^Health [36] may offer structured, computer-processable medical knowledge for personalized medicine in a manner similar to how they currently offer drug-drug interaction knowledge and structured order sets. Many of these companies, as well as others such as WebMD^® ^[37], also provide narrative educational content that could be expanded to help clinicians and patients better understand the appropriate use of genetic and genomic assays.

A second CDS infrastructure requirement is the standardization of the CDS information to be provided for various aspects of genomic and personalized medicine. For example, for the interpretation of gene expression analyses of cancer specimens, agreement could potentially be reached that CDS for this type of assay should include the recommended course of therapy, the expected survival of the patient on different therapies, and the evidence base underlying the assessment. Such standardization of CDS content across knowledge repositories will enable clinicians to more easily interpret and act on care recommendations regardless of the knowledge repository utilized for generating the CDS.

A third vital component of a national CDS infrastructure for genomic medicine will be the standardization of how relevant genomic and non-genomic patient data are represented in health information systems. Currently, there is significant heterogeneity in the way in which different clinical practices and health information systems capture and record electronic patient data, including genomic data [28]. Such heterogeneity is a significant problem because this variability can make it very difficult to take a CDS resource developed for one clinical setting and to re-leverage that resource in other settings [28]. To enable the efficient deployment of CDS capabilities, both the information models used to represent patient data and the terminologies used to identify concepts within those models will need to be standardized. Moreover, to enable truly personalized healthcare, existing clinical terminologies such as the Systematized Nomenclature of Medicine – Clinical Terms (SNOMED CT^®^) [38] and the Logical Observation Identifiers Names and Codes (LOINC^®^) [39] will need to be extended to encompass relevant concepts, such as genomically defined disease subtypes and genomic assay results. Moreover, if existing terminologies cannot be appropriately extended, new terminologies may need to be developed.

A fourth critical component of a national CDS infrastructure will be standard approaches for independent computer systems to leverage computer-processable genomic knowledge repositories and patient data to guide clinical decision making. Such approaches will need to include standard methods for identifying and accessing relevant knowledge resources, so that knowledge resources can be efficiently utilized by various clinical information systems. One cross-industry technology platform that could be leveraged for such knowledge sharing is Web services technology, which entails the provision of software capabilities through the standardized communication of extensible markup language (XML) messages over the Internet [40]. For example, knowledge repositories could provide a standard Web service interface for clients to search for knowledge resources of interest. Then, these repositories could provide a standard Web service interface for retrieving these resources in a standardized format (e.g., genetic risk assessment algorithms implemented in a standard rule expression format such as GELLO [41] or the Arden Syntax [42]). Alternatively, these repositories could provide a standard Web service interface for directly obtaining patient-specific inferences utilizing these resources. For example, a warfarin dosing module within a knowledge repository could accept relevant clinical and genetic parameters as Web service inputs and return the recommended dosing and rationale as the output.

Finally, a fifth critical component for a national CDS infrastructure will be a standard approach for locating and retrieving patient data from across disparate health information systems. Such standardized data retrieval is critical to ensure that all patient data relevant for a clinical decision can be efficiently and securely collected from the various healthcare organizations participating in the care of a given patient. Fortunately, the U.S. Health Insurance Portability and Accountability Act (HIPAA) permits such health information exchange among organizations that have entered into HIPAA business associate agreements, provided that appropriate privacy and security safeguards have been put into place [43].

### Relevant Resources

In designing, developing, and implementing a national CDS infrastructure for genomic and personalized medicine, it will be important to leverage available resources. Table 2 outlines several notable resources that could be leveraged for establishing the required CDS infrastructure components.

**Table 2:** CDS infrastructure needs and available resources

Infrastructure Need	Available Resources
Centrally managed repositories of computer-processable medical knowledge	Initiatives that can serve as models for creating authoritative knowledge on how genomic interventions should be used in clinical practice (e.g., U.S. Preventive Services Task Force [44], U.S. Evaluation of Genomic Applications in Practice and Prevention initiative [45])
	Centrally managed repositories of structured experimental data relevant to genomic medicine (e.g., PharmGKB for pharmacogenetic and pharmacogenomic experimental data and curated knowledge [46]; NCBI dbGaP for data from studies evaluating the interaction between genotypes and phenotypes [47]; and NCBI GEO for high-throughput, experimental genomic data [48])
	Various formalisms for representing medical knowledge in a computer-processable format [49,50] (e.g., Arden Syntax [42], GELLO [41], GLIF [52], PRO*forma *[53], SAGE [54], SEBASTIAN [55])
	Large-scale efforts at managing computer-processable medical knowledge (Intermountain Healthcare [56], Partners HealthCare [57])

Standardization of CDS information provided for specific aspects of genomic medicine	Standard mechanisms for defining CDS information to be provided in specified contexts (e.g., HL7 refined message information models [49], openEHR Archetypes [58], HL7 Decision Support Service semantic profiles [59])

Standardized representation of patient data	Standardized information models and terminologies for both genomic and traditional patient data [49,60] (e.g., HL7 data standards, including HL7 Clinical Genomics data standards [60] and emerging HL7 virtual medical record standard [62]; openEHR Archetypes [58]; SNOMED CT [38]; LOINC [39]; BSML [60]; MAGE-ML [63]; National Cancer Institute/caBIG Common Data Elements [61])
	Resources that enable mapping between different terminologies (e.g., Unified Medical Language System [65], National Cancer Institute Enterprise Vocabulary Services [66], HL7 Common Terminology Services standard [67])

Standard approaches for leveraging computer-processable medical knowledge	Various approaches for using computer-processable medical knowledge resources to provide automated CDS [31] (e.g., Arden Syntax [42], SAGE [54], PRODIGY [68], GLIF [69], SEBASTIAN [55], First DataBank Drug Information Framework [70], HL7 Decision Support Service [59])

Standard approach for locating and retrieving patient data	Standardized approaches for resolving patient identities across systems (e.g., HL7 Entity Identification Service draft standard [72] and corresponding OMG technical specification [73]) and for locating and retrieving patient data across systems (e.g., HL7 Retrieve, Locate, and Update Service draft standard [74] and corresponding OMG technical specification [75])
	Regional and national initiatives for secure health data exchange (e.g., U.K. National Health Service Connecting for Health [76], U.S. Nationwide Health Information Network prototypes [77], caBIG [78], Indiana Health Information Exchange [79])

All infrastructure components	Initiatives to share machine-processable medical knowledge (e.g., Morningside Initiative [80], U.S. federal CDS Collabatory [81])
	Efforts at specifying functional requirements of EHR systems (e.g., HL7 EHR System Functional Model standard [82], CCHIT EHR certification criteria [83])
	Efforts at coordinating the use of available health information technology standards (e.g., U.S. Health Information Technology Standards Panel [84], Integrating the Healthcare Enterprise [85])

BSML = Bioinformatic Sequence Markup Language; caBIG = cancer Biomedical Informatics Grid; CCHIT = Certification Commission for Healthcare Information Technology; dbGaP = database of Genotype and Phenotype; EHR = electronic health record; GEO = Gene Expression Omnibus; GLIF = Guideline Interchange Format; HL7 = Health Level 7; LOINC = Logical Observation Identifiers Names and Codes; MAGE-ML = microarray and gene expression markup language; NCBI = National Center for Biotechnology Information; OMG = Object Management Group; PharmGKB = Pharmacogenetics and Pharmacogenomics Knowledge Base; PRODIGY = Prescribing RatiOnally with Decision-support In General-practice studY; SAGE = Standards-Based Active Guideline Environment; SEBASTIAN = System for Evidence Based Advice through Simultaneous Transaction with an Intelligent Agent across a Network; SNOMED CT = Systematized Nomenclature of Medicine, Clinical Terms

First, with regard to the creation of centrally managed repositories of computer-processable medical knowledge, methods for generating authoritative knowledge on the appropriate use of genetics and genomics in clinical medicine have been developed by groups including the U.S. Preventive Services Task Force [44], which is sponsored by the U.S. Agency for Healthcare Research and Quality (AHRQ), and by the Evaluation of Genomic Applications in Practice and Prevention initiative [45], which is sponsored by the U.S. Centers for Disease Control and Prevention (CDC). These efforts and their associated methodologies could be applied systematically to emerging genetic and genomic interventions to provide definitive guidance on how they should be used in practice. Moreover, the creation of such guidance can be facilitated by the availability of centrally managed repositories of structured experimental data relevant to genomic medicine. For example, the Pharmacogenetics and Pharmacogenomics Knowledge Base (PharmGKB) offers both primary data and curated knowledge derived from pharmacogenetic and pharmacogenomic research studies [46]; the National Center for Biotechnology Information (NCBI)'s database of Genotype and Phenotype (dbGaP) provides access to data from studies evaluating the interaction between genotypes and phenotypes, including results of genome-wide association studies [47]; and NCBI's Gene Expression Omnibus (GEO) offers data from microarray experiments and other high-throughput genomic experiments [48].

Once the underlying medical knowledge on the appropriate use of genetic and genomic interventions is available, various formalisms are available for representing that knowledge in an appropriate computer-processable format [49,50]. These approaches include the Arden Syntax for Medical Logic Modules, which is a formalism for representing context-inclusive medical knowledge that is supported by several commercial EHR systems [42]. The Arden Syntax has been adopted as a standard by Health Level 7 (HL7), which is the leading standards development organization in healthcare IT internationally [51]. Another relevant HL7 standard is GELLO, which is an expression language based on the Object Constraint Language that can be used to represent data requirements and decision logic for computer-processable medical knowledge in a platform-independent manner [41]. Other available formalisms include the Guideline Interchange Format (GLIF) [52], PRO*forma *[53], and the Standards-Based Active Guideline Environment (SAGE) [54], which are context-inclusive formalisms for modeling clinical practice guidelines in a machine-processable manner. Also, the System for Evidence Based Advice through Simultaneous Transaction with an Intelligent Agent across a Network (SEBASTIAN) [55] provides a mechanism for accessing context-independent modules of machine-processable medical knowledge through a Web service interface.

In developing centralized CDS knowledge repositories for personalized medicine, additional resources that can be leveraged include the published descriptions of how Intermountain Healthcare in Utah [56] and Partners HealthCare in Boston [57] have developed enterprise-wide infrastructures for specifying, developing, and maintaining CDS assets. These knowledge management infrastructures are used operationally to support the collaborative specification, authoring, and maintenance of CDS assets that include narrative clinical guidelines, structured order sets, and machine-processable clinical decision rules [56,57]. These efforts are particularly useful in offering insights into how the efforts of large numbers of users, and in particular clinician domain experts with limited technical background, can be harnessed to inform the specification and implementation of computer-processable knowledge resources.

Second, with regard to the need for standards on the information included in CDS for specific aspects of genomic medicine, such information content can be standardized within HL7 as refined message information models, which are context-specific information models that derive from a unifying information model known as the HL7 Reference Information Model [49]. Such information content can also be represented as an openEHR Archetype [58]. Once defined, such information models could be designated as the output of a CDS knowledge resource such as a knowledge module in an HL7 Decision Support Service. Adopted as an HL7 draft standard in 2006, the Decision Support Service specification provides a standard interface for submitting patient data as the input and obtaining patient-specific inferences using one or more modules of computable medical knowledge [59].

Third, with respect to the need for standardized representation of patient data, standard information models and terminologies are available for representing both genomic and non-genomic patient data [49,60]. These available standards include HL7 data standards for traditional medical data such as problem lists, medication lists, and clinical encounter history [49]; HL7 data standards for family history information and genetic testing data developed by the HL7 Clinical Genomics Special Interest Group [60]; and data standards developed by the National Cancer Institute as a part of its cancer Biomedical Informatics Grid (caBIG^®^) initiative [61]. Also, there is an effort within the HL7 Clinical Decision Support Technical Committee to standardize a "virtual medical record" that defines the superset of patient data relevant for CDS [62].

The HL7 data standard for genetic testing incorporates clinically relevant subsets of bioinformatics data standards, including the bioinformatic sequence markup language (BSML) for sequence data [60] and the microarray and gene expression markup language (MAGE-ML) for gene expression data [63]. Of note, MAGE-ML complies with Minimum Information About a Microarray Experiment (MIAME) guidelines [64], which specify the minimal set of data on gene expression assays that should be included within an assay report to allow for proper interpretation. As for notable terminology resources, SNOMED CT is a clinical healthcare terminology with over 311,000 active, hierarchically organized clinical concepts [38], and LOINC is a major terminology of over 50,000 laboratory concepts that includes an expanding set of codes to identify genetic tests [39].

As additional resources for standardized patient data representation, resources are available to map concepts between terminologies in the case that data are stored using a terminology different than that used by a knowledge resource. Most notable among these resources is the National Library of Medicine (NLM)'s Unified Medical Language System (UMLS), which provides concept mappings across over a hundred terminologies [65]. Also, the National Cancer Institute Enterprise Vocabulary Services resource, which is based on the UMLS, provides additional support for several cancer-centric terminologies [66]. Moreover, the HL7 Common Terminology Services standard defines a standard interface for accessing such terminology services [67].

Fourth, with regard to the need for standard approaches for leveraging computer-processable medical knowledge, various methods have been developed for providing CDS using computer-processable medical knowledge resources [31]. In many of these methods, context-inclusive knowledge resources are loaded into a knowledge resource execution engine, which in turn interfaces with an EHR system or other point-of-care clinical information system. In this approach, the execution engine is triggered when an activation condition specified in the knowledge resource occurs. Then, the execution engine retrieves the required input data from the clinical information system, generates patient-specific assessments and recommendations, and communicate these inferences back to the user through the clinical information system [31]. This approach to leveraging machine-processable medical knowledge has been used by CDS approaches that include the Arden Syntax [42], SAGE [54], PRODIGY [68], and GLIF [69].

Methods have also been developed for providing CDS using context-independent knowledge resources. In these methods, the CDS execution engine typically provides an interface for submitting patient data as the input and obtaining patient-specific, computer-processable inferences as the output. In contrast to approaches for utilizing context-inclusive knowledge resources, this approach leaves contextual issues related to integrating the knowledge into the clinical information system up to the invoking information system (e.g., considerations of when to invoke the resource; how to retrieve the required patient data; and how to communicate the CDS results to the end-user) [31]. Methods that utilize this approach include SEBASTIAN [55], the First DataBank Drug Information Framework [70], and the HL7 Decision Support Service standard [59].

Fifth, with respect to the need for a standard approach for locating and retrieving relevant patient data across systems, relevant standards have been developed though the Healthcare Services Specification Project (HSSP), which is a joint initiative between HL7 and the Object Management Group (OMG) to develop interoperable service interface specifications for healthcare [71]. The HSSP has produced an HL7 draft standard and a corresponding OMG technical specification for an Entity Identification Service that resolves the identities of entities (e.g., patients) across systems [72,73]. Also, the HSSP has developed HL7 and OMG standards for a Retrieve, Locate, and Update Service that enables locating and retrieving patient data across systems [74,75]. The HL7 Decision Support Service [59] and HL7 Common Terminology Services [67] standards described earlier are also a part of this family of service interface standards. Moreover, potential approaches for secure, cross-organizational health data exchange have been explored by a number of efforts, including the U.K. National Health Service Connecting for Health [76] initiative, the U.S. Nationwide Health Information Network (NHIN) prototypes [77], caBIG [78], and the Indiana Health Information Exchange [79]. Some of the lessons learned from these prior efforts are discussed in the next section.

Furthermore, in designing and developing all of the infrastructure components, there is potential for synergy with two recently initiated, large-scale efforts at sharing CDS resources across institutions. One of these efforts, known as the Morningside Initiative, is a collaborative effort started in November 2007 to develop an open, Web-based repository of computer-processable medical knowledge [80]. The collaborators in this initiative are the American Medical Informatics Association (AMIA), Arizona State University, the Henry Ford Health System, the Veterans Health Administration, the Department of Defense, Kaiser Permanente, Partners HealthCare System, the U.S. Telemedicine and Advanced Technology Research Center, and Intermountain Healthcare. An important goal of this initiative is to develop a business model and funding streams for creating such a knowledge repository. A second large-scale effort at sharing CDS resources is the U.S. federal CDS Collaboratory, which is co-sponsored by the U.S. Office of the National Coordinator for Health Information Technology (ONCHIT), AHRQ, and the U.S. Department of Health and Human Services' Personalized Health Care Initiative [81]. The first meeting of the Collaboratory was held in March 2008, with representatives from nine U.S. federal agencies participating. The goal of the Collaboratory is to coordinate CDS efforts across the U.S. federal government in order to expedite the development and widespread adoption of effective CDS capabilities. This Collaboratory therefore provides an existing platform for coordinating government-sponsored CDS initiatives within the U.S.

Another type of resource that could be leveraged in establishing a national CDS infrastructure is efforts at specifying functional requirements of EHR systems, as CDS capabilities for personalized medicine could be incorporated within these requirement frameworks. A relevant resource in this regard is the HL7 EHR system functional model standard, which defines functional capabilities of EHR systems and conformance criteria for evaluating the availability of these capabilities within a given EHR system [82]. Furthermore, the Certification Commission for Healthcare Information Technology (CCHIT) is a nonprofit, private-sector organization which defines functional requirements for EHR systems and provides certification to those systems that fulfill all of these defined requirements [83]. Despite only being in existence since 2004, CCHIT already commands significant market influence, due to purchasers using CCHIT certification as an important criterion in system selection.

Finally, an important challenge to establishing a standards-based, semantically interoperable infrastructure for CDS is the fact that overlapping and oftentimes conflicting standards exist for the same purpose [49]. Thus, coordinating the selection and use of standards will be critical for establishing a CDS infrastructure for personalized medicine. A prominent effort that could be leveraged for this purpose is sponsored by the U.S. Health Information Technology Standards Panel (HITSP). HITSP is a public-private partnership founded in 2005 to promote interoperability by harmonizing and integrating health IT standards [84], and its current scope of work includes the coordination of existing standards to support personalized healthcare. Furthermore, Integrating the Healthcare Enterprise (IHE) is an initiative that was started in 1998 by the Radiological Society of North America and the Healthcare Information and Management Systems Society (HIMSS) to define how existing standards should be used to complete a particular healthcare task [85]. Similar to HITSP specifications, IHE specifications could be developed to define how standards should be used to provide CDS for personalized medicine.

### Lessons Learned from Prior Efforts

Beyond these specific resources just described, the effort to create a national CDS infrastructure for genomic and personalized medicine can leverage lessons learned from within the U.S. and abroad. In this section, we review some of these relevant insights.

First, with regard to how CDS needs to be delivered to catalyze desired improvements in clinical care, we previously identified 22 features of CDS interventions that had been suggested to be important for CDS effectiveness by at least three sources in the literature [29]. When evaluated against the results of 70 randomized controlled trials of electronic and non-electronic CDS interventions using multivariate regression analysis techniques, four of these features were found to be significantly associated with a CDS intervention's ability to lead to statistically and clinically significant improvements in clinical care: automatic provision of CDS as part of clinician workflow (p < 0.00001), provision of recommendations rather than just assessments (p = 0.0187), provision of CDS at the time and location of decision making (p = 0.0263), and the use of a computer to generate the CDS (p = 0.0294) [29]. Moreover, through randomized controlled trials comparing a CDS intervention against the same intervention augmented by a specific feature, direct evidence is available supporting the importance of providing CDS automatically as a part of clinician workflow [86]; providing CDS at the time and location of decision making [87]; supplementing point-of-care CDS with periodic user feedback on compliance with the care standards recommended by the CDS system [88]; and requiring clinicians to document a reason when deviating from the recommendations of a CDS system [89]. These insights from prior evaluations of CDS interventions can inform how patient-specific care recommendations relating to personalized medicine should be delivered to clinicians.

Moreover, when we analyzed existing approaches to the integration of machine-processable knowledge resources into clinical applications to enable CDS [31], we identified several general principles that are of relevance to establishing a national CDS infrastructure. First, in order to be widely useful, machine-processable medical knowledge resources should be comprehensive in content coverage and as easy as possible to leverage within diverse types of clinical information systems. Second, because machine-processable medical knowledge is currently encoded using a wide range of approaches, and because efforts at adopting a universal approach to knowledge representation have been unsuccessful to date despite a number of attempts, support should be provided for knowledge represented using different formalisms. Of note, the use of a common service interface such as the HL7 Decision Support Service interface [59] may allow machine-processable medical knowledge represented using different approaches to be leveraged in a consistent manner. Finally, experience with the market's positive but limited adoption of the HL7 Arden Syntax standard [42] indicates that standardization can greatly facilitate the market adoption of an approach to sharing CDS knowledge but is insufficient on its own to drive widespread adoption.

Finally, important insights are available from large-scale health IT initiatives that have been ongoing at regional and national levels. First, to establish a national health IT infrastructure capable of supporting advanced CDS, significant investments will be required. Launched in 2002, England's effort to establish a national health IT infrastructure, known as the National Programme for IT (NPfIT), was estimated to cost £12.4 billion (approximately $19 billion) in a 2008 report by England's National Audit Office [90]. In the U.S., an expert panel has estimated that establishing a national health IT infrastructure in the U.S. would cost $156 billion in capital investments over five years, with annual operating costs of $48 billion [91]. Second, large-scale health IT initiatives involving multiple healthcare organizations are likely to require significant government leadership and funding. In the U.S., efforts at exchanging health information across institutional boundaries have focused on the establishment of regional health information exchange networks known as regional health information organizations (RHIOs). While successful and financially viable RHIOs such as the Indiana Health Information Exchange [79] do exist, of 145 RHIOs surveyed in 2007, only 20 were at least of modest size and exchanging clinical data, and eight of these RHIOs were heavily dependent on grant funding to stay in operation [92]. These findings call into question whether a national health IT infrastructure can be established without significant and consistent funding from the federal government [92]. Similar conclusions can be drawn from a global survey of EHR implementation efforts published by HIMSS in August 2008 [93]. This report evaluated EHR implementation efforts in Germany, the Netherlands, Greece, England, Wales, Denmark, Norway, India, New Zealand, Malaysia, Hong Kong, Singapore, Israel, Canada, and the United States with regard to funding, governance, communication, and standardization and interoperability. Based on the lessons learned from these efforts around the globe, this report concludes that developing a national, interoperable EHR system in the U.S. will require the government to mandate the use of appropriate standards; provide adequate funding; establish clearer governance structures and reduce bureaucracy; and support improved communication among the relevant stakeholders [93]. As CDS depends to a large extent on the availability of a robust EHR infrastructure, active leadership and substantial funding from the federal government will likely be essential to the successful implementation of a national CDS infrastructure for genomic and personalized medicine.

### Recommendations

To facilitate the widespread and consistent practice of genomic and personalized medicine, we recommend that the following actions be taken.

• First, we recommend that healthcare stakeholders work together to develop a concrete action plan for developing and implementing the five critical components of a national CDS infrastructure as outlined in Table 1. We provide in the next section an initial action plan that could serve as the basis for developing a more detailed plan moving forward.

• Second, we recommend that efforts at creating a CDS infrastructure for genomic and personalized medicine be integrated with an overall effort to support decision making for all aspects of healthcare. Ultimately, genomic medicine will need to be integrated seamlessly within existing clinical contexts and clinical care pathways, such that genetically based medical interventions will be "just another aspect" of practicing modern medicine. Accordingly, the CDS infrastructure should fully support the use of both genomic and non-genomic patient data to guide clinical care. This integration of genomic medicine into clinical practice will also require the addressing of other barriers to clinical adoption, including the need for greater scientific evidence validating the clinical value and cost effectiveness of genetic and genomic assays [17-22], as well as the need for educating healthcare professionals regarding the appropriate use of genetics and genomics in practice [17,19,22,23].

• Third, we recommend leveraging existing resources to the greatest extent possible (Table 2).

• Fourth, we recommend that the national CDS infrastructure be aligned with the strategic objectives set forth in the *Roadmap for National Action on CDS*, which was commissioned by ONCHIT and generated by an expert AMIA task force in 2006 [28]. Of note, we have previously described how a national CDS infrastructure can fulfill these strategic objectives through the coordinated use of a number of the resources and standards listed in Table 2, including centrally managed knowledge repositories, Web-accessible software services with standard HL7 interfaces, and standard patient data models defined by HL7 and by other standards development organizations [94].

• Fifth, we recommend that the federal government lead the design, funding, and establishment of the CDS infrastructure. As noted earlier, large-scale, cross-organizational health IT efforts from the U.S. and abroad indicate that government leadership and funding are critical to the success of such efforts [92,93]. Moreover, as noted by Dr. Don Detmer, the President and Chief Executive Officer of AMIA, the federal government must lead the development of a national health information infrastructure (NHII), because (i) an NHII is a public good whose benefits accrue across many stakeholders; (ii) the federal government has a significant incentive to establish an NHII, as more effective use of health IT could reduce the enormous federal expenses associated with Medicare and Medicaid; and (iii) the private sector has insufficient centralized power or resources to lead such an effort [95].

• Sixth, given that less than 20% of U.S. clinicians currently utilize EHR systems in the ambulatory care setting [96], we recommend that the CDS infrastructure support both clinicians who already utilize EHR systems and clinicians who currently do not. To support clinicians who do not utilize EHR systems, care recommendations could potentially be provided using administrative claims data and standardized laboratory data. For example, laboratories could potentially utilize a patient's prior claims data to provide individually tailored interpretations of genetic and genomic test results.

• Seventh, given increasing recent interest in personal health records (PHRs) [97], including by major software vendors such as Microsoft^® ^[98] and Google^® ^[99], we recommend that the CDS infrastructure be designed to also support personalized medicine within the context of PHRs.

• Finally, when acquiring health information systems, we recommend that clinicians and healthcare organizations give preference to systems that provide robust CDS capabilities using both genomic and non-genomic patient data. We anticipate that such advanced CDS capabilities will be most readily available in information systems that take advantage of an emerging national CDS infrastructure for genomic and personalized medicine.

### Proposed Framework for Action

We present here a proposed initial framework for moving forward. We hope that this proposal will serve as the catalyst for discussions and action that ultimately lead to the establishment of a national CDS infrastructure for genomic and personalized medicine.

As the first step, the U.S. federal CDS Collaboratory could be clearly established as the organization that will lead the effort to design, develop, and implement a national CDS infrastructure supportive of personalized medicine. Besides ONCHIT, AHRQ, and the Personalized Health Care Initiative, federal agencies that could play central roles in this process include the Department of Health and Human Services, the National Institutes of Health, the Food and Drug Administration, the CDC, the Veterans Health Administration, the Military Health System, the Health Resources and Services Administration, and the Centers for Medicare & Medicaid Services (CMS). Crucially, substantial funding will need to be secured by members of the Collaboratory on an ongoing basis in order for this effort to succeed. While led by federal agencies, the Collaboratory should also work closely with non-governmental organizations, including HITSP, CCHIT, HL7, academic medical centers, healthcare delivery organizations, and commercial health IT vendors.

Next, the Collaboratory could lead the definition of desired CDS capabilities for personalized medicine in terms of a large number of detailed use cases organized under common patterns, and these use cases could be used to define CCHIT EHR certification criteria. At the same time, the Collaboratory could provide funding to relevant domain experts in government, academia, and industry to develop relevant standards within HL7 and to specify how new and existing standards should be utilized within the framework of HITSP. Moreover, CMS could provide financial incentives for healthcare organizations to obtain access to targeted CDS capabilities, similar to how CMS currently provides a bonus to clinicians for using electronic prescribing systems in their practices [100].

Beyond leading the specification of the requirements of clinical information systems supportive of personalized medicine, the Collaboratory could internally develop and/or fund the external development of HITSP-compliant approaches to fulfilling the CCHIT certification criteria in relation to personalized medicine. For example, the NLM could lead an effort within the federal government to develop freely accessible knowledge repositories for personalized medicine in a manner similar to how it has developed the UMLS terminology resource [65]. As another potential approach, the federal government could license knowledge resources from academic medical centers, professional medical associations, and/or commercial entities so that these resources can be freely used by healthcare organizations throughout the nation. This type of arrangement already has a precedent in that the NLM currently licenses SNOMED CT for approximately $6 million per year so that anyone in the U.S. may use it free of charge [101]. Moreover, these approaches to supporting the adoption of CDS capabilities for personalized medicine could be extended beyond the creation of knowledge repositories. For example, the federal government could provide internally developed or licensed software components and support services to facilitate the creation of secure health information exchange networks, so that CDS systems can provide accurate advice even when patients receive care across organizational boundaries.

Obviously, the extent to which these steps can be taken by the federal government depends critically on the amount of funding that can be secured for supporting health IT in general and CDS specifically. While we recognize that budgets are likely to remain tight within the federal government for some time, we are hopeful for progress given President Obama's many public statements that health IT is a key potential driver for more effective and cost-effective healthcare that is worthy of federal support [102].

### Call to Seize the Limited Window of Opportunity

Given that genomic medicine is still in its early stages [103], some may argue that it is premature to establish a national CDS infrastructure for genomic and personalized medicine. However, it is precisely because genomic medicine is in its infancy that there is a significant opportunity for directing the growth and development of a robust infrastructure. Currently, only a limited amount of genetic test data is stored in operational health information systems, and CDS for genomic medicine is still rare even in research settings. If we do not establish a standards-based infrastructure expeditiously, however, health information systems will likely store increasing amounts of genomic data using idiosyncratic approaches, and efforts at providing widespread, effective CDS for genomic medicine will be severely hindered by the presence of entrenched information systems that lack interoperability. To avoid this modern-day Tower of Babel, we must act now while the window of opportunity remains open.

## Summary

Genomic medicine offers great promise for achieving a more personalized and effective approach to disease prevention, diagnosis, and therapy. To fulfill the full promise of genomic and personalized medicine, however, a national CDS infrastructure will be needed to guide the appropriate use and interpretation of new genomic assays. Core components of this required infrastructure include centrally managed repositories of computer-processable medical knowledge; standardization of the CDS information to be provided for various aspects of genomic and personalized medicine; standardized representation of genomic and non-genomic patient data; standard approaches for leveraging genomic knowledge repositories and patient data to guide clinical care; and a standard approach for retrieving relevant patient data across health information systems. In order to meet this important and emerging need, we recommend the following: healthcare stakeholders should come together to design and develop the five components of the national CDS infrastructure identified in this manuscript; efforts at creating a national CDS infrastructure for genomic medicine should be integrated with an overall effort to create a national CDS infrastructure for all aspects of healthcare; existing resources should be leveraged as much as possible; the national CDS infrastructure should be aligned with the strategic objectives of the U.S. *Roadmap for National Action on CDS*; the federal government should lead the establishment of the infrastructure; the infrastructure should support clinicians even if they do not have access to fully functional EHR systems; the infrastructure should include support for PHRs; and when acquiring health information systems, healthcare organizations should select systems that provide robust CDS capabilities using both genomic and non-genomic patient data. Because genomic medicine is still in its early stages of development, a limited window of opportunity exists for establishing a robust, standards-based, and interoperable CDS infrastructure before healthcare organizations and health IT vendors develop entrenched, institution-specific approaches to the problem that lack interoperability. We therefore call upon the various healthcare stakeholders in the U.S. and elsewhere to work expeditiously towards a national CDS infrastructure for genomic and personalized medicine, as the establishment of this infrastructure will be critical for enabling a future in which each patient's unique genetic profile is routinely considered to optimize health and healthcare.

## Competing interests

HFW and GSG have no competing interests. KK and DFL are co-owners of Kedays LLC, which holds intellectual property rights, including rights to a pending patent application, for a CDS technology known as SEBASTIAN. This technology has been licensed to Religent, Inc. for commercial development through a Small Business Technology Transfer grant from the U.S. National Library of Medicine (grant R42LM009051). SEBASTIAN is capable of providing its capabilities through the HL7 Decision Support Service interface mentioned in Table 2. KK, DFL, Kedasys LLC, Religent, Inc., and Duke University may benefit financially if products utilizing the SEBASTIAN technology are commercially successful.

## Authors' contributions

All authors contributed to the conception and design of the manuscript, and KK drafted the manuscript. All authors contributed to the critical revision of the manuscript, and all authors read and approved the final manuscript.

## Authors' information

KK, MD, PhD is a member of HL7 and the project lead of the HL7 Decision Support Service project. DFL, MD, PhD, MS was a member of the task force of the American Medical Informatics Association (AMIA) that produced the *Roadmap for National Action on Clinical Decision Support*. HFW, PhD is the Director of the Institute for Genome Sciences & Policy (IGSP) at Duke University. He is also Vice Chancellor for Genome Sciences at Duke University Medical Center and a former member of the Advisory Committee on Genetics, Health and Society for the Secretary of the U.S. Department of Health and Human Services. GSG, MD, PhD is the Director of the IGSP's Center for Genomic Medicine. The views expressed in this manuscript are those of the authors alone and do not necessarily represent the views of HL7, AMIA, Duke University, the National Human Genome Research Institute, or the National Institutes of Health.

## Pre-publication history

The pre-publication history for this paper can be accessed here:

http://www.biomedcentral.com/1472-6947/9/17/prepub
